# Furfural tolerance and detoxification mechanism in *Candida tropicalis*

**DOI:** 10.1186/s13068-016-0668-x

**Published:** 2016-11-18

**Authors:** Shizeng Wang, Gang Cheng, Chijioke Joshua, Zijun He, Xinxiao Sun, Ruimin Li, Lexuan Liu, Qipeng Yuan

**Affiliations:** 1 State Key Laboratory of Chemical Resource Engineering, College of Life Science and Technology, Beijing University of Chemical Technology, West Room 314, Science and Technology Building, No. 15 North Third Ring East Road, Chaoyang District, Beijing, 100029 People’s Republic of China; 2 Deconstruction Division, Joint BioEnergy Institute, Emeryville, CA 94608 USA

**Keywords:** *Candida tropicalis*, Furfural tolerance, Furfural detoxification, Alcohol dehydrogenase 1

## Abstract

**Background:**

Current biomass pretreatment by hydrothermal treatment (including acid hydrolysis, steam explosion, and high-temperature steaming) and ionic liquids generally generate inhibitors to the following fermentation process. Furfural is one of the typical inhibitors generated in hydrothermal treatment of biomass. Furfural could inhibit cell growth rate and decrease biofuel productivity of microbes. *Candida tropicalis* is a promising microbe for the production of biofuels and value-added chemicals using hemicellulose hydrolysate as carbon source. In this study, *C. tropicalis* showed a comparable ability of furfural tolerance during fermentation. We investigated the mechanism of *C. tropicalis*’s robust tolerance to furfural and relevant metabolic responses to obtain more information for metabolic engineering of microbes for efficient lignocellulose fermentation.

**Results:**

*Candida tropicalis* showed comparable intrinsic tolerance to furfural and a fast rate of furfural detoxification. *C. tropicalis*’s half maximal inhibitory concentration for furfural with xylose as the sole carbon source was 3.69 g/L, which was higher than that of most wild-type microbes reported in the literature to our knowledge. Even though furfural prolonged the lag phase of *C. tropicalis*, the final biomass in the groups treated with 1 g/L furfural was slightly greater than that in the control groups. By real-time PCR analysis, we found that the expression of *ADH1* in *C. tropicalis* (*ctADH1*) was induced by furfural and repressed by ethanol after furfural depletion. The expression of *ctADH1* could be regulated by both furfural and ethanol. After the disruption of gene *ctADH1*, we found that *C. tropicalis*’s furfural tolerance was weakened. To further confirm the function of *ctADH1* and enhance *Escherichia coli*’s furfural tolerance, *ctADH1* was overexpressed in *E. coli* BL21 (DE3). The rate of furfural degradation in *E. coli* BL21 (DE3) with pET-ADH1 (high-copy plasmid) and pCS-ADH1 (medium-copy plasmid) was increased by 1.59-fold and 1.28-fold, respectively.

**Conclusions:**

*Candida tropicalis* was a robust strain with intrinsic tolerance to inhibitor furfural. The mechanism of furfural detoxification and metabolic responses were identified by multiple analyses. Alcohol dehydrogenase 1 was confirmed to be responsible for furfural detoxification. *C. tropicalis* showed a complex regulation system during furfural detoxification to minimize adverse effects caused by furfural. Furthermore, the mechanism we uncovered in this work was successfully applied to enhance *E. coli*’s furfural tolerance by heterologous expression of *ctADH1*. The study provides deeper insights into strain modification for biofuel production by efficient lignocellulose fermentation.

**Electronic supplementary material:**

The online version of this article (doi:10.1186/s13068-016-0668-x) contains supplementary material, which is available to authorized users.

## Background

Lignocellulosic biomass is an attractive and promising substrate source for the production of biofuels and value-added chemicals. Pretreatment is a primary and key step for efficient and economic bioconversion of lignocellulosic biomass [1]. However, during pretreatment of lignocellulose by acid hydrolysis, steam explosion, and high-temperature steaming [2–5], several groups of inhibitors for fermentation are generated, such as furfural, phenolic compounds, and acetic acid [6–8]. Furfural, derived from the dehydration of pentose, is one of the primary inhibitors in hemicellulose hydrolysate. Furfural negatively affected specific growth rate of cells [9] and acted as inhibitor to glycolytic enzymes [7]. Moreover, furfural could increase the toxicity of acetate in yeasts [10] and the toxicity of phenols in *Escherichia coli* [11, 12]. In *Saccharomyces cerevisiae*, furfural has been shown to cause an accumulation of reactive oxygen species and cellular damage to mitochondria, vacuoles, actin, and nuclear chromatin [13]. In *Candida tropicalis*, growth and xylitol fermentation could also be inhibited by furfural [14].

To reduce the inhibition caused by furfural, recent research has been focused on the mechanisms of tolerance and detoxification in microbes. However, resistance phenotypes usually involve complicated multi-genic regulations among stress responses [15]. Silencing of oxidoreductase genes increased furfural tolerance in *E. coli* LY180 [16]. Overexpression of propanediol oxidoreductase and transhydrogenase improved the growth of *E. coli* in the presence of furfural [17, 18]. Recent research showed that overexpression of alcohol dehydrogenases (encoded by *ADH*), transcription activator Msn2, oxidative stress regulator Yap1, and glucose-6-phosphate dehydrogenase, which were confirmed by transcriptional analysis, proteomic analysis, and disruption library screening, could improve furfural tolerance in yeast [19–22]. In *S. cerevisiae*, furfural could be converted into furfuryl alcohol by NADH-dependent alcohol dehydrogenase 1 [23–25]. Co-expression of transaldolase 1 and alcohol dehydrogenase 1 in recombinant xylose-fermenting *S. cerevisiae* improves the production of ethanol and xylitol in xylose medium in the presence of furfural [26]. However, furfuryl alcohol is not stable, and the detection of this compound is complicated for its polymerization [27] and degradation [28].

In our previous study, *C. tropicalis* was used to produce xylitol from hemicellulose hydrolysate [2]. The metabolic responses caused by complex inhibitors (including furfural, acetic acid, and phenol) in *C. tropicalis* have been studied by a gas chromatography/mass spectrum-based metabolomics approach [29]. We also found that *C. tropicalis* showed faster furfural detoxification rate in xylose medium than in glucose medium [30]. However, *C. tropicalis*’s resistance to furfural has not been evaluated. The mechanism of furfural tolerance and metabolic responses to furfural were still unknown for *C. tropicalis*. In this study, we found that *C. tropicalis* showed a comparable intrinsic tolerance to furfural by half maximal inhibitory concentration (IC_50_) analysis. To provide deeper insights into the mechanisms of furfural tolerance and metabolic responses, we investigated the expression of key genes involved in ethanol production using quantitative real-time PCR (qRT-PCR). We found that *ADH1* from *C. tropicalis* (*ctADH1*) played a key role in the resistance to furfural, and furfural triggered a series of complex responses in *C. tropicalis* to eliminate furfural and maintain redox balance. Furthermore, *E. coli*’s furfural tolerance was successfully enhanced by heterologous expression of *ctADH1*. The mechanism of *C. tropicalis*’s furfural tolerance elucidated in this work will provide useful information for metabolic engineering of efficient strains for lignocellulose fermentation.

## Methods

### Strains, medium, and plasmids

Wild-type *C. tropicalis* (Strain No. 2.1776, China General Microbiological Culture Collection Center, China) was employed in fermentation. In our previous study, we obtained uracil auxotroph *C. tropicalis* YE (*ura3*/*ura3*) derived from wild-type *C. tropicalis* by chemical mutagenesis. *C. tropicalis* YE was used as the host strain for gene disruption. *E. coli* DH5α was used as the host strain for plasmid construction and propagation. *E. coli* BL21 (DE3) was used for protein expression. When needed, ampicillin and kanamycin were added into the medium with the final concentration of 100 μg/mL. The details of strains used in this study are depicted in Additional file 1: Table S1.

Preculture medium was used for inoculation. Xylose medium was used for toxicity test, tolerance test, IC_50_ analysis, transcriptional analysis, and HPLC analysis of *C. tropicalis*. The fermentation was carried out at 30 °C and 200 rpm in 250-ml flask for *C. tropicalis*. Yeast extract peptone dextrose (YPD) medium, yeast nitrogen base without amino acids (YNB) medium, YNB-uracil (YNB-URA) medium, and YNB-URA-5-fluoroorotic acid (YNB-URA-5FOA) medium were used for genetic manipulation of *C. tropicalis*. Luria–Bertani (LB) medium was used for inoculants, propagation, and genetic manipulation of *E. coli*. Modified minimal basal salts (M9) medium was used for furfural degradation experiment of *E. coli*. The fermentation was carried out at 37 °C and 200 rpm in 250-ml flask for *E. coli*. pCS27 and pETDuet-1 are high- and medium-copy plasmids, respectively. They were both used for protein expression. The details of plasmids used in this study are depicted in Additional file 1: Table S1. The constituents of medium were described in Additional material.

### Growth curves, IC_50_, and cell viability test under furfural stress

In furfural tolerance tests, furfural of 0, 1, 3, 5, 7, and 9 g/L was added at the beginning of fermentation. The optical density at 600 nm (OD_600_) of the biomass was measured by UV spectrophotometer (Shimadzu UV-2450, China) at 600 nm after dilution. The growth curves of *C. tropicalis* were determined by OD_600_. The values of IC_50_ were calculated by Graphpad Prism probit analysis (GraphPad Software, USA).

Methylene blue staining was used to evaluate *C. tropicalis*’s cell viability in the presence of furfural. In cell viability analysis, furfural of 1, 3, 5, 7, and 9 g/L was added into the culture at mid-exponential phase (6 h after inoculation), when the cells showed the most significant metabolic responses to stress. Cells were harvested and dropped onto a clean glass slide. Methylene blue solvent (0.1%) was added to the cells. After having been stained for 30 min, cells were observed by optical microscopy (Olympus CX 23, Japan). The dead cells were stained dark blue, while the living cells were transparent.

### Fermentation liquor analysis and redox balance analysis

In fermentation experiments, furfural of 3, 5, and 7 g/L was added to the fermentation at mid-exponential phase (6 h after inoculation) in furfural-treated groups. After centrifugation, samples were filtered through a 0.22-μm filter. The supernatant was diluted before being analyzed by high-performance liquid chromatography (HPLC). The analysis of xylose, xylitol, and furfural were performed with a Hitachi HPLC system (Hitachi Chromaster, Japan). They were separated by an Aminex HPX-87H column (Bio-Rad, USA) at 45 °C and detected by a refractive index detector. 5 mM H_2_SO_4_ was used as mobile phase at a flow rate of 0.6 mL/min. The ratio of NADH/NAD^+^ in the samples were extracted and measured by NADH/NAD^+^ Assay Kit (BioAssay Systems, USA).

### RNA extraction, cDNA synthesis, and qRT-PCR

TRIzol Reagent (Invitrogen, USA) was used to extract total RNA from cells pellets by liquid nitrogen grinding. RNA samples were quantified by SmartSpec plus (Bio-rad). RNA quality was determined by electrophoresis. We employed reverse transcription with random hexamer primed reactions by M-MLV First Strand cDNA Synthesis Kit (Omega Bio-Tek, China). The cDNA was stored at −20 °C. Relative quantification of cDNA was performed by Perfectstart SYBR Green qPCR master mix (Omega Bio-Tek) on a Mastercycler Realplex2 cycler (Eppendorf, Germany). The primers used in qRT-PCR are listed in Additional file 1: Table S2. Samples were analyzed in triplicates, with negative controls in each assay. The expression of related genes was calculated relative to the expression of *ACTIN* by the comparative C_T_ method. *T* tests were performed by SPSS 19.0 (SPSS Inc., USA).

### Construction of *ctADH1* disruption cassettes, transformation, and sensitivity experiment

Gene disruption of *C. tropicalis* was performed by homologous recombination [31, 32]. The *URA3* gene (with promoter and terminator, GenBank accession number AB006207.1) was amplified by PCR from the genomic DNA of wild-type *C. tropicalis* with primers URA3-F and URA3-R [31]. The *ctADH1* gene (NCBI Reference Sequence: XM_002546589.1) and *ctADH1m* (the middle part of *ctADH1*, 0.55 kb) were amplified by PCR from the genomic DNA of uracil auxotroph *C. tropicalis* YE with primers ADH1-F-ADH1-R and ADH1m-F-ADH1m-R, respectively. *hisG1* fragments (1.1 kb) and *hisG2* fragments (1.1 kb) were amplified by PCR from the plasmid pCUB6 [33] with two sets of primers, hisG1-F-hisG1-R and hisG2-F-hisG2-R, respectively. *ctADH1* and *ctADH1m* were inserted into T-Vector pMD19 (Simple), and the plasmid was designated Ts-ADH1 and Ts-ADH1m, respectively.

ADH1-Ts-ADH1 was amplified by PCR from Ts-ADH1 with Ts-Ar-F and Ts-Ar-R. URA3 digested by *Sac*I and *Pst*I was ligated with ADH1-Ts-ADH1 digested by *Sac*I and *Pst*I. The resulting plasmid was designated Ts-ADH1-URA3. *hisG1* and *hisG2* were inserted into Ts-ADH1-URA3 one by one, resulting in plasmid Ts-AUH and Ts-AUHH. ADH1-hisG1-URA3-hisG2-ADH1, which was used as the first disruption cassette, was amplified from Ts-AUHH with ADH1-F and ADH1-R. ADH1m-Ts-ADH1m was amplified by PCR from Ts-ADH1m with Ts-Amr-F and Ts-Amr-R. URA3 digested by *Sac*I and *Pst*I, was ligated with ADH1m-Ts-ADH1m digested by *Sac*I and *Pst*I. The resulting plasmid was designated Ts-AmU. ADH1m-URA3-ADH1m, which was used as the second disruption cassette, was amplified from Ts-ADH1m-URA3 with ADH1m-F and ADH1m-R.

*Candida tropicalis* was transformed using the LiCl method [34]. The first disruption cassette ADH1-hisG1-URA3-hisG2-ADH1 was transformed into *C. tropicalis* YE. The transformants (*C. tropicalis* T1) selected on YNB plates were confirmed by PCR. The transformants with *URA3* marker were spread on YNB-URA-5FOA plates. The *URA3* pop-out mutants (*C. tropicalis* T2 and *C. tropicalis* T3) were selected from the 5-FOA-resistant colonies using PCR. The second disruption cassette was transformed into *C. tropicalis* T2, and the resulting cells were selected by YNB plate. The transformants (*C. tropicalis* T4) were confirmed by PCR. The primers used in this study are listed in Additional file 1: Table S2.

To verify the furfural sensitivity of *C. tropicalis*, cells of *C. tropicalis* T4, T3, T2, and YE were inoculated into 15-ml glass tubes containing 5 ml of YPD and 3 g/L furfural. The tubes were incubated for 10 h at 30 °C and 200 rpm.

### Heterologous expression of *ctADH1* and in vivo furfural degradation in *E. coli*

*ctADH1* was subcloned into pETDuet-1 by *Sac*I and *Kpn*I, and into pCS-27 by *Sal*I and *Sma*I, resulting plasmid pET-ADH1 and pCS-ADH1. The primers used in this study are listed in Additional file 1: Table S2. To evaluate alcohol dehydrogenase 1’s activity of degrading furfural, the plasmid pET-ADH1, pETDuet-1, pCS-ADH1, and pCS-27 were transformed into *E. coli* BL21 (DE3). The resultant transformants (*E. coli* PE, *E. coli* PC, *E. coli* PEA, and *E. coli* PCA) were inoculated in 5 mL M9 medium and cultured at 37 °C. The overnight cultures were inoculated into 50 mL M9 medium. The medium was cultured at 37 °C. When OD_600_ reaching 0.6, the cultures were induced by 0.25 mM of β-d-1-thiogalactopyranoside (IPTG) at 30 °C. Furfural of 1 g/L was added into the culture 6 h after inoculation. The concentration of furfural was measured by HPLC. The expression of *ctADH1* was confirmed by SDS-PAGE analysis [35].

## Results and discussion

### Tolerance and in situ detoxification of *C. tropicalis* under furfural stress

**Fig. 1:**
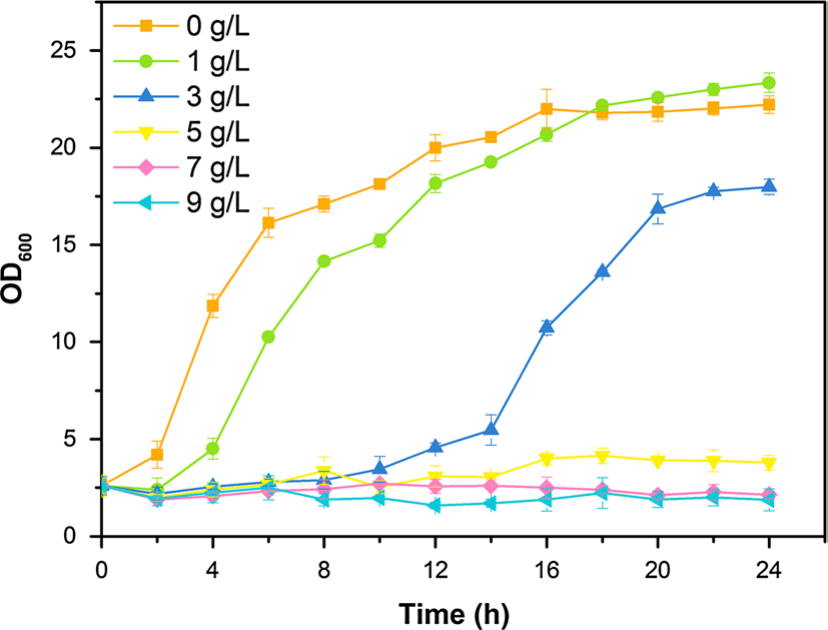
Growth profiles during fermentation in the presence of furfural. Furfural of 0, 1, 3, 5, 7, and 9 g/L was added into xylose medium at the beginning of fermentation. The *error bars* represent the standard deviation calculated from triplicates

In this study, furfural tolerance and cell viability of furfural in *C. tropicalis* were evaluated by growth curves, IC_50_, and methylene blue staining. To determine the growth inhibition caused by furfural, furfural of 0, 1, 3, 5, 7, and 9 g/L was added into the medium at the beginning of fermentation. The lag phase of *C. tropicalis* in growth curves was significantly prolonged by furfural (Fig. 1). Interestingly, even though 1 g/L furfural prolonged the lag phase of *C. tropicalis*, the final biomass produced in the medium containing 1 g/L furfural was slightly greater than that in the control (Fig. 1). It was reported that furfural of 10 mM (approximately 1 g/L) slightly increased *Thermoanaerobacter pseudethanolicus*’s cell yield by 11% [35]. Furfural could replace glycerol as an electron sink and allow less carbon loss to byproducts and more carbon distribution towards biomass synthesis [36]. However, *C. tropicalis* could hardly grow up in the presence of 5 g/L furfural at the beginning of fermentation (Fig. 1). *C. tropicalis*’s IC_50_ (Table 1) for furfural was calculated by growth curves (Fig. 1). *C. tropicalis*’s IC_50_ for furfural was 3.69 g/L at 24 h. To our knowledge, the IC_50_ of *C. tropicalis* was higher than most wild-type microbes reported in the literature (Table 1). Although the direct comparisons of IC_50_ between different organisms are difficult due to the differences in the culture conditions, our results suggest that *C. tropicalis* possesses a comparable tolerance to furfural.

**Table 1:** IC_50_ (g/L) for furfural in different microorganisms

Organism	IC_50_ for furfural	Carbon source (w/v)	Time (h)	Temp (°C)	Ref.
*C. tropicalis* YE	3.69	10% xylose	24	30	This study
*C. shehatae*	1.02	2% xylose	32	26	[36]
*Pichia stipitis*	0.85	2% xylose	32	26	[36]
*Zymomonas. mobilis*	1.93	2% glucose	24	30	[36]
*S. cerevisiae* CBS 1200	0.51	2% glucose	24	26	[36]
*S. cerevisiae* NSI 113	2	1% glucose	48	30	[37]
Mixed culture from anaerobic sludge	1.05	4% glucose	48	37	[38]
*Bacillus coagulans*	2.5–5	5–10% glucose	24	50	[39]
*Caldicellulosiruptor saccharolyticus*	1–2	1% glucose	16, 40	72	[40]
*Thermotoga neapolitana*	2–4	1% glucose	16, 40	80	[40]
*E. coli*	2.4, 2.9	5% xylose	24, 48	37	[11]
*Thermoanaerobacter pseudethanolicus*	3	0.7% glucose	12	65	[41]
*Thermoanaerobacter pseudethanolicus*	3–4	0.7% glucose	24	65	[41]

Methylene blue staining was used to evaluate cell viability. Furfural of 1, 3, 5, 7, and 9 g/L was added at mid-exponential phase (6 h after inoculation). Cell viability test (Table 2; Additional file 1: Figure S1) by methylene blue staining showed that quite few cells died at an early stage of furfural treatment (0.5 h after addition of furfural). With the increase of treatment time, the proportion of dead cells increased until depletion of furfural. Furfural caused more cell death 1 h and 2.5 h after furfural treatment. However, the proportion of living cells increased 5 h after furfural treatment. In the presence of 1, 3, 5, and 7 g/L furfural, *C. tropicalis* could still reproduce, and living cells were dominant as shown by the cell viability test (Table 2; Additional file 1: Figure S1), showing comparable robustness to furfural. However, 9 g/L furfural caused more than 50 and 90% cells dead 1 and 2.5 h after furfural treatment (Table 2; Additional file 1: Figure S1), respectively. The result indicated that 9 g/L furfural was lethal for *C. tropicalis*. The result of methylene blue staining was also used to determine the appropriate concentration of furfural used in the following experiments, since furfural of high concentrations would cause cell death directly [19]. After cell death, the analysis of fermentation liquor would provide poor information, and RNA in cells would degrade soon. Therefore, it was determined that furfural of 3, 5, and 7 g/L was suitable for analysis of the transcription and fermentation liquor.

**Table 2:** Ratio of dead cells to total cells counted by the result of methylene blue staining

Time after furfural treatment	Ratio of dead cells to total cells				
	1 g/L furfural	3 g/L furfural	5 g/L furfural	7 g/L furfural	9 g/L furfural
30 min	1.6% (2/126)	3.3% (5/152)	2.3% (5/218)	4.2% (9/213)	11.7% (24/206)
1 h	1.5% (2/129)	3.3% (5/151)	7% (10/142)	25% (45/180)	61.5% (40/65)
2.5 h	2.3% (2/870)	5.6% (10/178)	18.2% (18/99)	25.8% (63/244)	95.1% (136/143)
5 h	1% (1/104)	1% (1/101)	14.9% (23/154)	21% (35/167)	93.3% (70/75)

The results of growth curves, IC_50_, and cell viability test indicated that *C. tropicalis* exhibited comparable tolerance under furfural stress. The proportion of living cells increased 5 h after furfural treatment, suggesting that the stress caused by furfural was reduced 5 h after furfural treatment. The reduced furfural stress was probably due to the degradation and detoxification of furfural.

In fermentation liquor analysis, when adding furfural of 3, 5, and 7 g/L into the culture at mid-exponential phase (6 h after inoculation), the rate of furfural degradation reached 3, 3.33, and 2.80 gL^−1^h^−1^, respectively. Furfural degradation rate was calculated by the following equation:

$$ {\text{Furfural degradation rate}}\;({\text{gL}}^{ - 1} {\text{h}}^{ - 1} )= \frac{{C_{f} }}{{T_{2} - T_{1} }}, $$

where *C*_*f*_, *T*_1_, and *T*_2_ refer to initial furfural concentration, time of furfural addition, and time of furfural depletion, respectively.

**Fig. 2:**
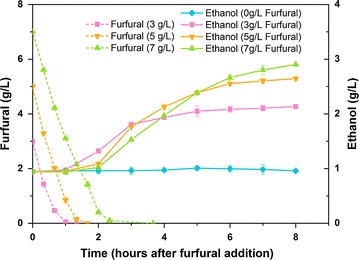
Furfural and ethanol profiles during fermentation in the presence of furfural. Furfural of 0, 3, 5, and 7 g/L was added into xylose medium at mid-exponential phase. The *error bars* represent the standard deviation calculated from triplicate experiments

Furfural was all depleted in 3 h after addition of furfural at mid-exponential phase (Fig. 2). Degradation rate of furfural in the groups treated by 5 g/L furfural was even faster than that treated by 3 g/L furfural. It suggested that furfural of medium concentration (5 g/L) might induce faster and stronger metabolic responses, and result in faster degradation rate, than furfural of low concentration (3 g/L). However, furfural of high concentration (7 g/L) was too toxic to induce efficient metabolic responses. As shown in Fig. 2, the degradation of furfural was followed by ethanol production. As soon as the furfural was depleted, ethanol concentration started to increase. In addition, the concentration of ethanol produced in the fermentation increased with the concentration of furfural added in the medium. It was proposed that the depletion of furfural triggered ethanol production. The levels of ethanol in furfural-treated groups showed more than twofold increase compared to those in control groups (1 g/L). The production of ethanol was probably relevant to the time of furfural degradation and the concentration of furfural added in the medium.

From above results, we presumed that the enzymes which were induced by furfural could also catalyze the production of ethanol. In other words, the enzymes involved in ethanol biosynthesis might play a key role in metabolic responses to furfural or furfural detoxification. In *C. tropicalis*, the enzyme directly involved in ethanol biosynthesis was alcohol dehydrogenase 1 (encoded by *ctADH1*). To validate the hypothesis, qRT-PCR, redox balance analysis, gene knock-out experiment, and overexpression of alcohol dehydrogenase 1 were carried out in this study.

### Metabolic responses of *C. tropicalis* under furfural stress

**Fig. 3:**
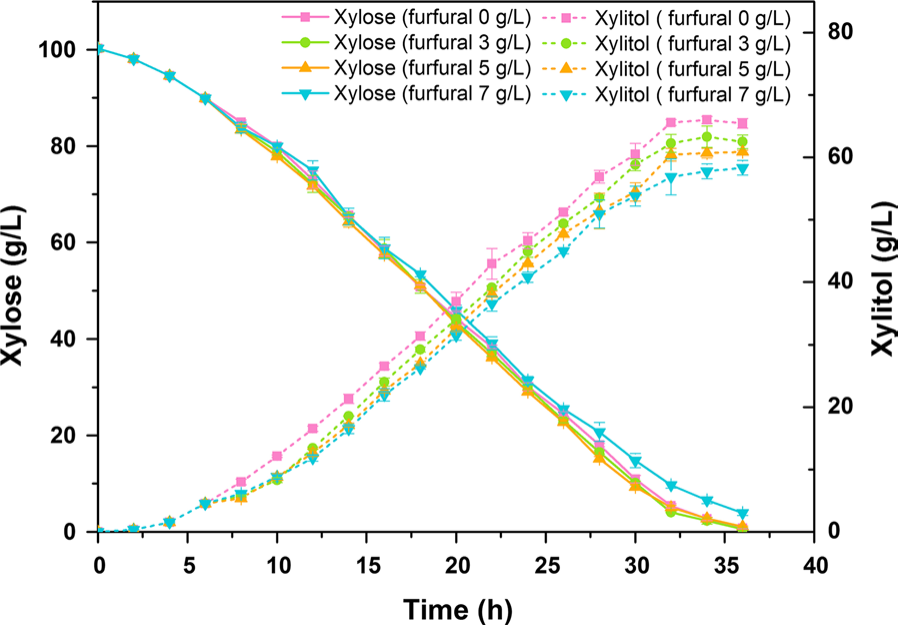
Xylose and xylitol profiles during fermentation in the presence of furfural. Furfural of 0, 3, 5, and 7 g/L was added into xylose medium at mid-exponential phase. The *error bars* represent the standard deviation calculated from triplicate experiments

In *C. tropicalis*, xylose is firstly reduced into xylitol by xylose reductase which uses both NADPH and NADH as cofactors [42]. Subsequently xylitol dehydrogenase almost exclusively uses NAD^+^ as cofactor in the oxidation of xylitol into xylulose [43]. After phosphorylation, xylulose is utilized by the pentose phosphate pathway to provide a carbon source and energy for cells. Furfural of various concentrations showed different effects on the consumption of xylose, when furfural was added 6 h after inoculation. Furfural of lower concentration (3 and 5 g/L) did not significantly inhibit the consumption rate of xylose (Fig. 3), indicating that the rate of xylose transportation or xylose metabolism was not affected by furfural of low concentrations. Low concentrations of furfural even slightly promoted xylose consumption rate at 28 h. After addition of furfural, xylitol accumulation was considerably decreased and delayed (Fig. 3). The more furfural was added, the less xylitol was accumulated. Xylitol concentration in the groups treated by 7 g/L furfural was decreased by 15% compared to the control. These results indicated that the proportion of xylitol consumed for providing energy and intermediate metabolites increased, and less xylitol was accumulated after furfural treatment. Hyung-Min et al. reported that the *E. coli* strain with greater furfural detoxification rate also showed a greater sugar consumption rate [44]. Ask et al. found that furfural stress could induce energy-requiring repair mechanism, and the energy charge was slightly higher after addition of furfural compared to the control [45]. Our previous study also showed that complex inhibitors could enhance TCA cycle to provide more energy and metabolic intermediates for inhibitor tolerance [29]. However, when adding furfural of high concentration (7 g/L) into the medium, xylose consumption rate was severely inhibited (Fig. 3), indicating that the xylose transportation or xylose metabolism was blocked by furfural of high concentrations. The flux of the pentose phosphate pathway would decrease accordingly.

**Fig. 4:**
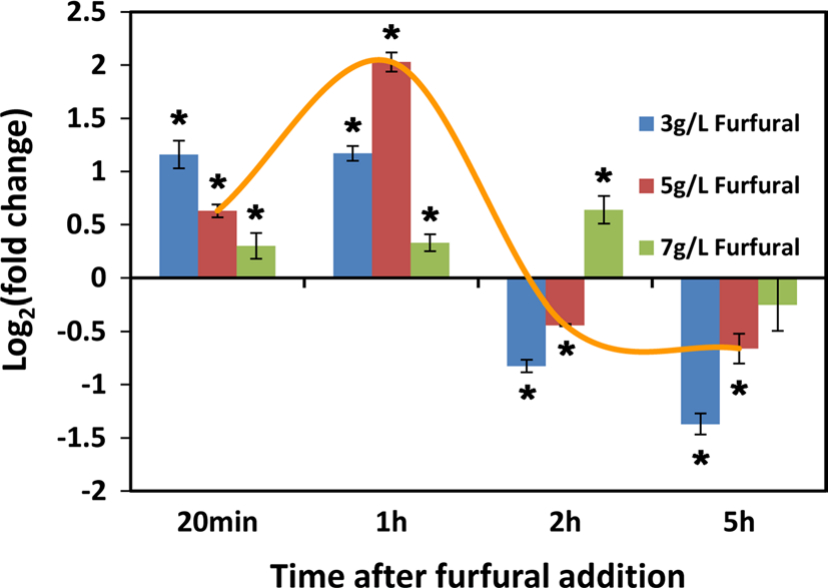
Transcription analysis of *ctADH1* by qRT-PCR after furfural treatment. Furfural of 0, 3, 5, and 7 g/L was added into xylose medium at mid-exponential phase. The *error bars* represent the standard deviation calculated from triplicate experiment. *p < 0.05 compared with the control

In the groups treated by furfural of 3, 5, and 7 g/L, the transcription of gene *ctADH1* showed the same transcriptional pattern (Fig. 4). The transcription of *ctADH1* was increased by furfural treatment. After furfural was totally degraded, *ctADH1* showed a decreased transcriptional level. This indicated that the expression of *ctADH1* in *C. tropicalis* was remarkably induced by furfural as metabolic responses, and after the depletion of furfural the transcription of *ctADH1* was immediately restrained (Fig. 4). Thus, we presumed that alcohol dehydrogenase 1 (encoded by *ctADH1*) was relevant to furfural degradation.

**Fig. 5:**
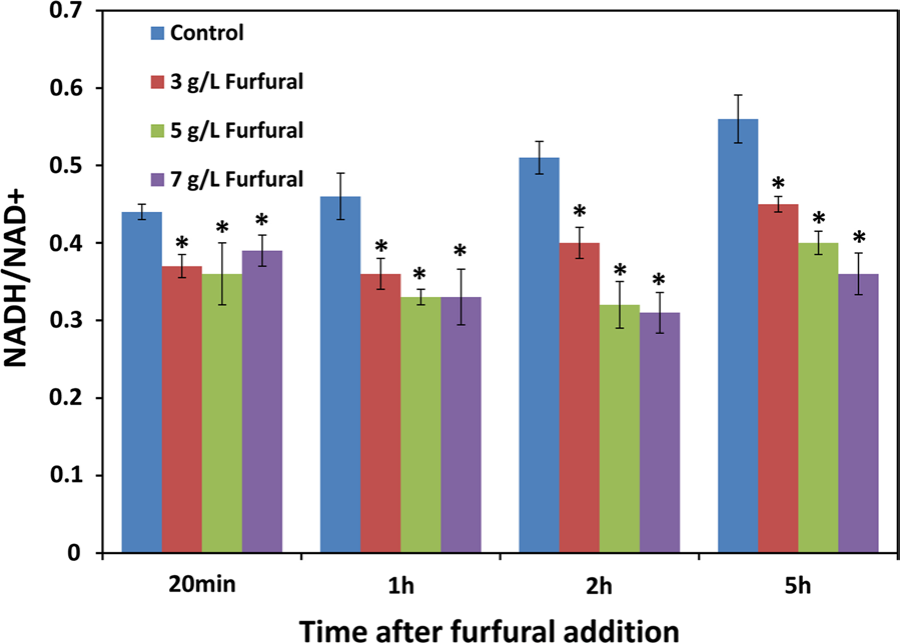
Ratio of intracellular NADH/NAD^+^ in the presence of furfural. Furfural of 0, 3, 5, and 7 g/L was added into xylose medium at mid-exponential phase. The *error bars* represent the standard deviation calculated from triplicate experiments. *p < 0.05 compared with the control

In the present study, the NADH/NAD^+^ ratio was used to determine the change of redox balance. NADH/NAD^+^ ratio in *C. tropicalis* decreased after furfural treatment compared with the control (Fig. 5). Even after the depletion of furfural, NADH/NAD^+^ ratio in *C. tropicalis* with furfural was still lower than those in the control, suggesting that the process of furfural degradation probably consumed the coenzyme NADH, and the disturbance of redox balance caused by furfural was long-lasting.

It was reported that the NADH/NAD^+^ ratio was decreased by furfural degradation in *S. cerevisiae* as a result of NADH consumption [19]. The results provided more evidence to prove that furfural was probably reduced by alcohol dehydrogenase 1 with NADH as coenzyme in *C. tropicalis*. It was also reported that the reduction of furfural to furfuryl alcohol might be catalyzed by alcohol dehydrogenases with NADH as a cofactor in *S. cerevisiae*, and glycolysis was possibly activated by furfural to provide more NADH as is required for the reduction of furfural [19, 46]. Furfuryl alcohol was unstable and less toxic than furfural.

### Role of *ctADH1* played in furfural detoxification

To confirm the function of *ctADH1*, we used homologous recombination system to disrupt the gene *ctADH1* [31]. Since *C. tropicalis* was diploid, cassettes were integrated into the two copies of *ctADH1* fragment region in the *C. tropicalis* genome one by one. The site-specific insertion of the transformants was confirmed by PCR (Additional file 1: Figure S2).

To verify the function of *ADH1* in *C. tropicalis*, cells of *C. tropicalis* T4, T3, T2, and YE were inoculated into YPD medium containing 3 g/L furfural. *C. tropicalis* T4 could hardly grow up in YPD medium containing 3 g/L furfural after the knockout of *ctADH1* (Additional file 1: Figure S3). Furfural tolerance of *C. tropicalis* T3 and *C. tropicalis* T2 was also weakened after the destruction of *ctADH1* in the first chromosome (Additional file 1: Figure S3). However, the parent strain *C. tropicalis* YE showed better growth in the presence of furfural compared with *ctADH1* disruption strains. This result proved that *C. tropicalis*’s robustness and tolerance to furfural were related to alcohol dehydrogenase 1. Laadan et al. found that HMF and furfural could be reduced by NADH-dependent alcohol dehydrogenase 1 in *S. cerevisiae* [25].

To further confirm the function of *ctADH1*, alcohol dehydrogenase 1 was overexpressed in *E. coli*. *ctADH1* was inserted into the vector pETDuet-1 and pCS-27. The insertion was confirmed by DNA sequencing of the PCR product. The expression of alcohol dehydrogenase 1 in *E. coli* PEA and *E. coli* PCA was confirmed by SDS-PAGE analysis (Additional file 1: Figure S4).

The rate of furfural degradation and detoxification was an indication of tolerance to furfural. When cultured in M9 medium, *E. coli* PEA and *E. coli* PCA showed much stronger ability of furfural degradation (Additional file 1: Figure S5A, B) than the control. The rate of furfural degradation in *E. coli* BL21 (DE3) with pET-ADH1 and pCS-ADH1 was increased by 1.59-fold and 1.28-fold compared to the control, respectively, indicating that overexpression of *ctADH1* improved *E. coli*’s ability of detoxicating furfural. The ability of degrading furfural in *E. coli* increased with the level of *ctADH1* expression. This result confirmed that alcohol dehydrogenase 1 from *C. tropicalis* could degrade furfural. Hasunuma also found that overexpression of NADH-dependent alcohol dehydrogenase 1 could improve ethanol fermentation in the presence of furfural in *S. cerevisiae* [24]. Moreover, heterologous expression of *ctADH1* in *E. coli* resulted in stronger furfural detoxification, which was a promising application of *ctADH1* for the production of lignocellulosic biofuel.

### The mechanism of furfural detoxification in *C. tropicalis*

**Fig. 6:**
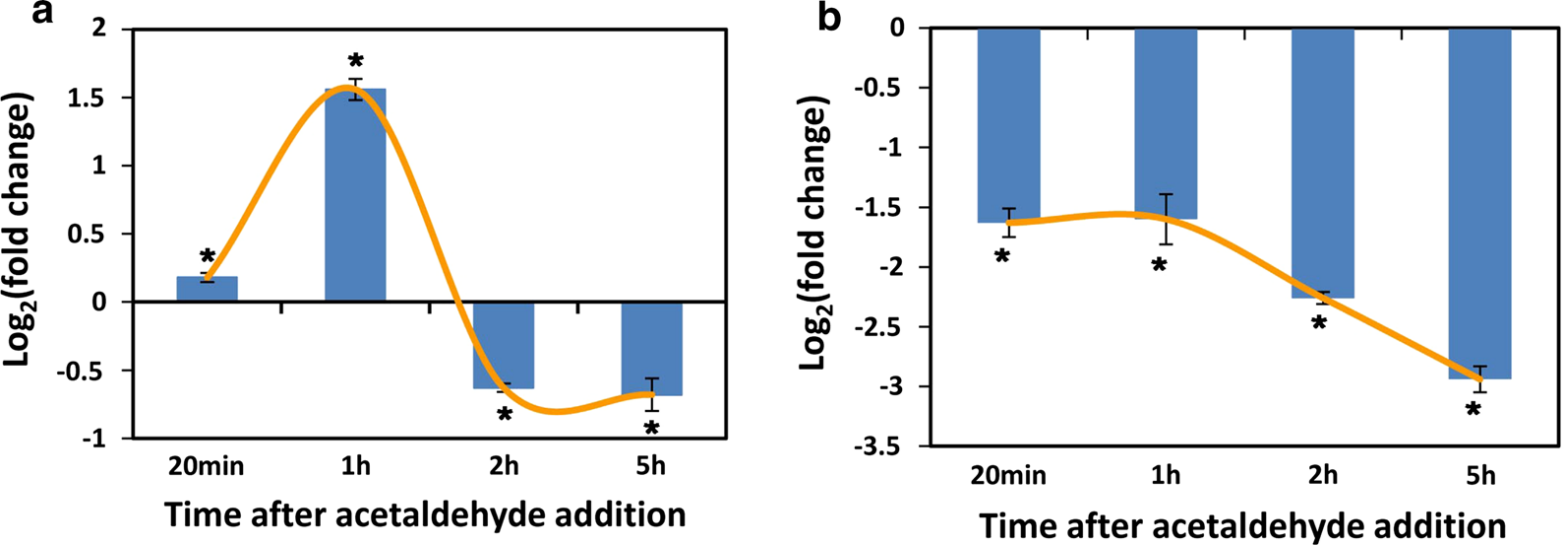
Acetaldehyde and ethanol regulated the transcription of *ctADH1*. **a** Transcription analysis of *ctADH1* after acetaldehyde addition; **b** Transcription analysis of *ctADH1* after ethanol addition. Acetaldehyde or ethanol of 2 g/L was added into the xylose medium culture at mid-exponential phase. The *error bars* represent the standard deviation calculated from triplicate experiments. *p < 0.05 compared with the control

To determine the mechanism of induction of alcohol dehydrogenase 1 by furfural, the effect of acetaldehyde on transcription of *ctADH1* was analyzed by qRT-PCR. Interestingly, the transcriptional response of *ctADH1* in *C. tropicalis* treated by acetaldehyde (2 g/L) showed similar pattern to that in *C. tropicalis* treated by furfural (Figs. 4, 6a). Alcohol dehydrogenase 1 is known for catalyzing the transformation from acetaldehyde to ethanol [47]. The gene *ctADH1* was up-regulated after acetaldehyde addition. However, the production of ethanol would consume reducing power. After the depletion of extra acetaldehyde, the transcriptional level of *ctADH1* decreased to restrain ethanol production and maintain redox balance. Furfural has the same functional group-aldehyde as acetaldehyde, which might lead to the same signal transduction and the same active site in alcohol dehydrogenase 1. Furfural and acetaldehyde caused the same transcriptional responses of *ctADH1* in *C. tropicalis*, which further supported the hypothesis that furfural was degraded by alcohol dehydrogenase 1 in *C. tropicalis*. However, after depletion of furfural, alcohol dehydrogenase 1 still remained in the cell. Subsequently, ethanol was produced by alcohol dehydrogenase 1, leading to the increase of ethanol level in the medium (Fig. 2). As enzyme, alcohol dehydrogenase 1 was not stable in the cell. After the degradation of alcohol dehydrogenase 1, the production of ethanol reached stationary phase. To determine the effect of ethanol on the transcription of *ctADH1*, ethanol of 2 g/L was added into the medium 6 h after inoculation. qRT-PCR analysis showed that the transcription of *ctADH1* was down-regulated 20 min, 1, and 2 h after addition of ethanol (Fig. 6b). The down-regulation of *ctADH1* after furfural depletion could help cells to avoid the loss of energy and carbon source, since alcohol dehydrogenase 1 is no more required for furfural detoxification. The repression of *ctADH1* could also minimize ethanol production and NADH consumption, making up for the imbalance of NADH/NAD^+^ ratio caused by furfural.

**Fig. 7:**
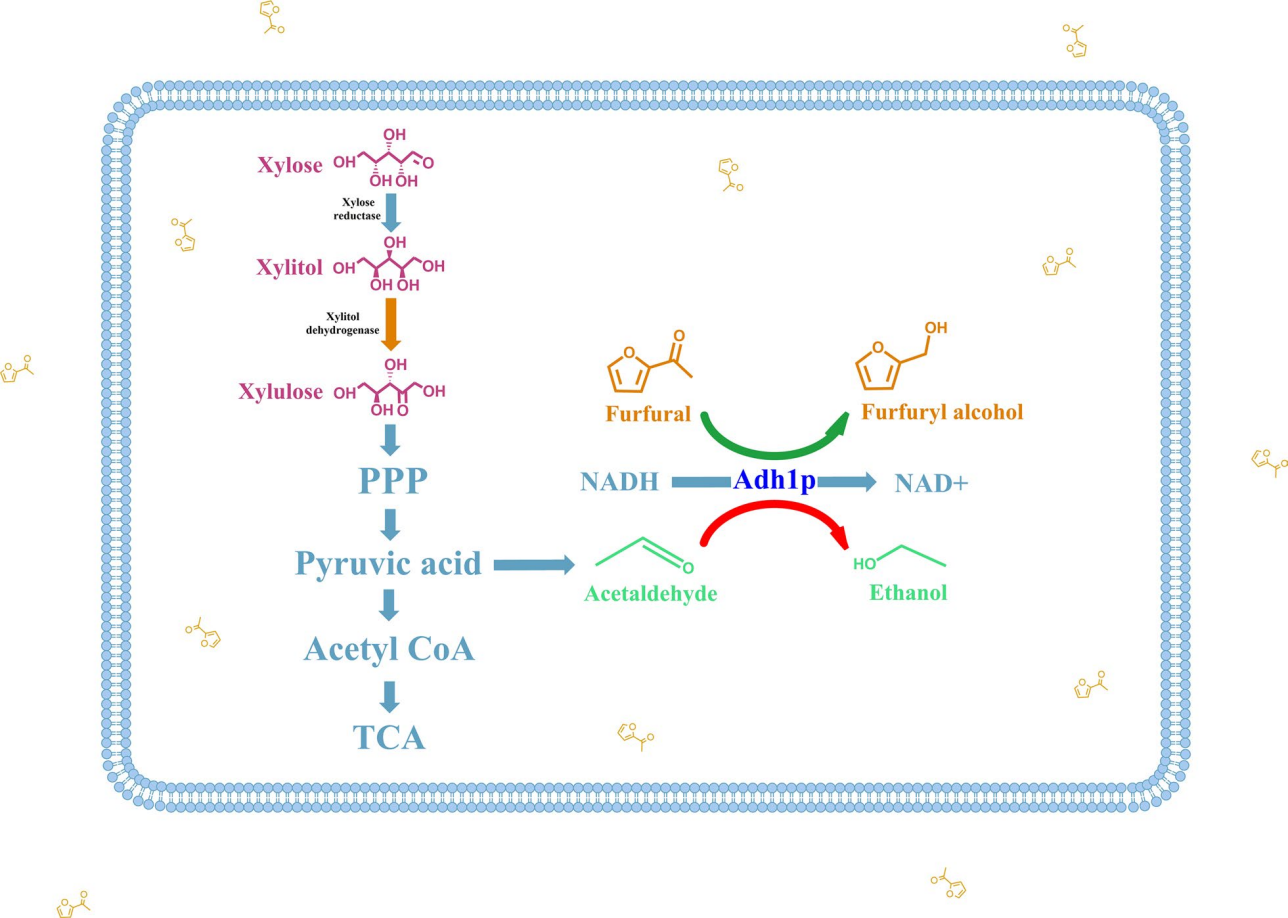
Mechanism of furfural tolerance and metabolic responses to furfural in *C. tropicalis*. Alcohol dehydrogenase 1 catalyzes the reduction of furfural and acetaldehyde with NADH as coenzyme. The* green arrow* represents that the transcription of *ctADH1* is induced by furfural, and furfural could be degraded by alcohol dehydrogenase 1 (encoded by *ctADH1*). The process of furfural degradation causes the imbalance of NADH/NAD^+^ ratio (Fig. 5). After furfural depletion, alcohol dehydrogenase 1 left in cells unavoidably produces ethanol. The* red arrow* represents that the transcription of *ctADH1* is repressed by ethanol to decrease ethanol production and NADH consumption. The repression of *ctADH1* contributes to maintain the redox balance

The transcription of *ctADH1* was regulated by both furfural and ethanol (Fig. 7). The induction of *ctADH1* was triggered by furfural to decrease toxic stress, which was followed by ethanol production. After furfural depletion, the transcription of *ctADH1* was repressed by ethanol to produce less ethanol and maintain redox balance. The regulation system of *ctADH1* minimized the toxic stress of furfural and oxidative stress caused by furfural detoxification and ethanol production.

## Conclusions

In this study, we found that *C. tropicalis* was a robust strain with intrinsic tolerance to the pretreatment inhibitor furfural. The IC_50_ for furfural with xylose as the sole carbon source was 3.69 g/L, which was higher than most wild-type microbes reported in the literature to our knowledge. Mechanisms of furfural detoxification and metabolic responses in the main pathways were revealed by multiple analyses. Alcohol dehydrogenase 1 was confirmed responsible for furfural detoxification. Furfural triggered the induction of *ctADH1* to degrade furfural. After furfural depletion, the transcription of *ctADH1* was repressed by ethanol to maintain redox balance (Fig. 7). The regulation system gives *C. tropicalis* comparable tolerance to furfural. Understanding furfural tolerance in *C. tropicalis* at molecular level is of significant importance for biofuel fermentation. The study provides valuable insights into tolerance enhancement and strain modification for efficient lignocellulose fermentation.

## Additional file

**Additional file 1: Table S1.** Strains and plasmids used in this study. **Table S2.** Primers used in this study. **Figure S1.** Furfural tolerance test evaluated by methylene blue staining. Furfural of 1, 3, 5, 7, and 9 g/L was added into the culture at mid-exponential phase. After having been stained by Methylene blue solvent 30 min, cells were observed and photoed by optical microscope. **Figure S2.** PCR confirmation of the specific integration in sequential *ctADH1* disruption. Lane M, DNA makers; Lane A, PCR from *C. tropicalis* YE genome with primers ADH1-F and ADH1-R resulting in the band of 1.2 kb (ADH1); Lane B, PCR from *C. tropicalis* Y1 genome with primers ADH1-F and ADH1-R resulting in the band of 1.2 and 4.3 kb (ADH1 and ADH1a-HisG-URA3-HisG-ADH1b); Lane C, PCR from *C. tropicalis* Y2 genome with primers ADH1-F and ADH1-R resulting in the band of 1.2 and 1.7 kb (ADH1 and ADH1a-HisG-ADH1b); Lane D, PCR from *C. tropicalis* Y4 genome with primers ADH1-F and ADH1-R resulting in the band of 1.7 and 2.9 kb (ADH1a-HisG-ADH1b and ADH1-URA3). **Figure S3.** Sensitivity experiment of *C. tropicalis* T4, T3, T2, and YE (parent strain). Cells of *C. tropicalis* T4, T3, T2, and YE were inoculated into 5 ml YPD medium containing 3 g/L furfural. The cultures were incubated for 10 h at 30 °C. **Figure S4.** SDS-PAGE of alcohol dehydrogenase 1 expressed in *E. coli*. Lane M, protein molecular weight markers (Thermo Scientific, #26610, USA); Lane A, *E. coli* PCA cells after IPTG induction; Lane B, *E. coli* PCA cells before IPTG induction; Lane C, *E. coli* PC cells without IPTG induction; Lane D, *E. coli* PEA cells after IPTG induction; Lane E, *E. coli* PEA cells before IPTG induction; Lane F, *E. coli* PE cells without IPTG induction. Molecular weight of alcohol dehydrogenase 1 is around 43 kDa. **Figure S5.** In vivo furfural degradation in recombined *E. coli*. (A) Furfural degradation of *E. coli* PCA (with pCS-ADH1) and PC (with pCS-27) in M9 medium; (B) Furfural degradation of *E. coli* PEA (with pET-ADH1) and PE (with pETDuet-1) in M9 medium.

## Abbreviations

IC_50_: half maximal inhibitory concentration
IPTG: β-d-1-thiogalactopyranoside
qRT-PCR: quantitative real-time PCR
YPD: yeast extract peptone dextrose
YNB-5FOA: yeast nitrogen base without amino acids-5-fluoroorotic acid
LB: Luria–Bertani
M9 medium: minimal basal salts medium
OD: optical density
HPLC: high-performance liquid chromatography
PPP: pentose phosphate pathway
