# Supplementary material for: Furfural tolerance and detoxification mechanism in Candida tropicalis
Source: Biotechnol Biofuels. 2016 Nov 18;9:250. doi: 10.1186/s13068-016-0668-x (PMC5116146; doi:10.1186/s13068-016-0668-x)
Supplement: Supplementary file 1 — Additional file 1: Table S1. Strains and plasmids used in this study. Table S2. Primers used in this study. Figure S1. Furfural tolerance test evaluated by methylene blue staining. Furfural of 1, 3, 5, 7, and 9 g/L was added into the culture at mid-exponential phase. After having been stained by Methylene blue solvent 30 min, cells were observed and photoed by optical microscope. Figure S2. PCR confirmation of the specific integration in sequential ctADH1 disruption. Lane M, DNA makers; Lane A, PCR from C. tropicalis YE genome with primers ADH1-F and ADH1-R resulting in the band of 1.2 kb (ADH1); Lane B, PCR from C. tropicalis Y1 genome with primers ADH1-F and ADH1-R resulting in the band of 1.2 and 4.3 kb (ADH1 and ADH1a-HisG-URA3-HisG-ADH1b); Lane C, PCR from C. tropicalis Y2 genome with primers ADH1-F and ADH1-R resulting in the band of 1.2 and 1.7 kb (ADH1 and ADH1a-HisG-ADH1b); Lane D, PCR from C. tropicalis Y4 genome with primers ADH1-F and ADH1-R resulting in the band of 1.7 and 2.9 kb (ADH1a-HisG-ADH1b and ADH1-URA3). Figure S3. Sensitivity experiment of C. tropicalis T4, T3, T2, and YE (parent strain). Cells of C. tropicalis T4, T3, T2, and YE were inoculated into 5 ml YPD medium containing 3 g/L furfural. The cultures were incubated for 10 h at 30 °C. Figure S4. SDS-PAGE of alcohol dehydrogenase 1 expressed in E. coli. Lane M, protein molecular weight markers (Thermo Scientific, #26610, USA); Lane A, E. coli PCA cells after IPTG induction; Lane B, E. coli PCA cells before IPTG induction; Lane C, E. coli PC cells without IPTG induction; Lane D, E. coli PEA cells after IPTG induction; Lane E, E. coli PEA cells before IPTG induction; Lane F, E. coli PE cells without IPTG induction. Molecular weight of alcohol dehydrogenase 1 is around 43 kDa. Figure S5. In vivo furfural degradation in recombined E. coli. (A) Furfural degradation of E. coli PCA (with pCS-ADH1) and PC (with pCS-27) in M9 medium; (B) Furfural degradation of E. coli PEA (with pET-ADH1) and PE (wi [file 13068_2016_668_MOESM1_ESM.docx]

**Furfural tolerance and detoxification mechanism in Candida tropicalis**

Shizeng Wang^1^, Gang Cheng^1^, Chijioke Joshua^2^, Zijun He^1^, Xinxiao Sun^1^, Ruimin Li^1^, Lexuan Liu^1^, Qipeng Yuan^1^*

^1^State Key Laboratory of Chemical Resource Engineering, College of Life Science and Technology, Beijing University of Chemical Technology, Beijing 100029, PR China

^2^Deconstruction Division, Joint BioEnergy Institute, Emeryville, California 94608, USA

* Correspondence to Professor Qipeng Yuan

West Room 314, Science and Technology Building, Beijing University of Chemical Technology, No. 15 North Third Ring East Road, Chaoyang District, Beijing 100029, PR China

E-mail address: yuanqp@mail.buct.edu.cn

Tel: +86 10 6442 7610; Fax: +86 10 64437610

**Medium used in this study**

Preculture medium is composed of 20 g/L xylose, 20 g/L glucose, 10 g/L yeast extract, 5 g/L KH2PO4, 0.5 g/L MgSO4 and 3 g/L (NH4)2HPO4.

Xylose medium is composed of 100 g/L xylose, 8 g/L yeast extract, 4 g/L KH2PO4, 0.5 g/L MgSO4 and 3 g/L (NH4)2HPO4).

YPD medium is composed of 20 g/L peptone, 10 g/L yeast extract, and 20 g/L glucose.

YNB medium is composed of 6.7 g/L yeast nitrogen base without amino acids, 20 g/L glucose and 10 g/L (NH4)2HPO4).

YNB-URA medium is composed of 6.7 g/L yeast nitrogen base without amino acids, 20 g/L glucose, 10 g/L (NH4)2HPO4, and 0.06 g/L uracil.

YNB-URA-5FOA medium is composed of 6.7 g/L yeast nitrogen base without amino acids, 20 g/L glucose, 10 g/L (NH4)2HPO4, 0.8 g/L 5-fluoroorotic acid and 0.06 g/L uracil.

LB medium is composed of 10 g/L peptone, 5 g/L yeast extract and 10 g/L NaCl.

Modified M9 medium is composed of 10 g/L glucose, 6 g/L Na2HPO4, 0.5 g/L NaCl, 3 g/L KH2PO4, 1g/L NH4Cl, 0.12 g/L MgSO4, 0.01 g/L CaCl2, 5 g/L yeast extract, and 2 g/L MOPS

| Strain/plasmid | Genotype | Referenceor source |
| --- | --- | --- |
| Strains |  |  |
| *C. tropicalis* | Wild-type ( CGMCC 2.1776) *URA3*/*URA3* *ADH1*/A*DH1* | CGMCC |
| *C.* *tropicalis* YE | *ura3*/*ura3* *ADH1*/*ADH1* | This study |
| *C. tropicalis* T1 | *ura3*/*ura3* *adh1*△::*HUH*/*ADH1* | This study |
| *C. tropicalis* T2  *C. tropicalis* T3 | *ura3*/*ura3* *adh1*△::*HisG*/*ADH1* | This study |
|  | *ura3*/*ura3* *adh1*△::*HisG*/*ADH1* | This study |
| *C. tropicalis* T4 | *ura3*/*ura3* *adh1*△::*HisG*/ *adh1*△::*URA3* (ADH1 disrupted) | This study |
| *E. coli* DH5α | Host for plasmid construction and propagation | TransGen |
| *E. coli* BL21 (DE3) | Host for protein expression | TransGen |
| *E. coli* PE | *E. coli* BL21 (DE3) with pETDuet-1 | This study |
| *E. coli* PC | *E. coli* BL21 (DE3) with pCS-27 | This study |
| *E. coli* PEA | *E. coli* BL21 (DE3) with pET-ADH1 | This study |
| *E. coli* PCA | *E. coli* BL21 (DE3) with pCS-ADH1 | This study |
| Plasmids |  |  |
| pETDuet-1 | pT7, PBR322 ori, Ampr | Novagen |
| pCS-27 | PLlacO1, P15A ori, Kanr | [[1](#_ENREF_1)] |
| pET-ADH1 | pETDuet-1 alcohol dehydrogenase 1 from *C. tropicalis* | This study |
| pCS-ADH1 | pCS-27 alcohol dehydrogenase 1 from *C. tropicalis* | This study |
| T-Vector pMD19 (Simple) | Cloning vector, Ampr | Takara |
| Ts-ADH1 | T-Vector pM19 (Simple) *ADH1* | This study |
| Ts-ADH1m | T-Vector pM19 (Simple) *ADH1m* | This study |
| Ts-ADH1-URA3 | T-Vector pM19 (Simple) *ADH1m URA3* | This study |
| Ts-AUH | T-Vector pM19 (Simple) *ADH1a HisG URA3 ADH1b* | This study |
| Ts-AUHH | T-Vector pM19 (Simple) *ADH1 HisG URA3 HisG ADH1b* | This study |
| Ts-AmU | T-Vector pM19 (Simple) *ADH1ma URA3 ADH1mb* | This study |

**Table S1 Strains and plasmids used in this study**

**Table S2 Primers used in this study.**

| Primer | Sequence | Restriction site(s) |
| --- | --- | --- |
| URA3-F | GGGAAAGAGCTCTTGGCGGGATCCTACTCTAACGACGGGTACAAC | SacI BamHI |
| URA3-R | GGGAAACTGCAGTTGGCGGTCGACACCCGATTTCAAAAGTGCAGA | PstI  SalI |
| ADH1-F | ATGCATGCACAATTCTCAAAAT |  |
| ADH1-R | TTATTTAGCAGTGTCCAAAACG |  |
| ADH1m-F | TGGCACGGTGACTGGCCATT |  |
| ADH1m-R | TCTTGACAGCACCGACAATG |  |
| hisG1-F | GGGAAAGAGCTCCTTCCAGTGGTGCATGAACGC | SacI |
| hisG1-R | GGGAAAGGATCCGCTGTTCCAGTCAATCAGGGT | BamHI |
| hisG2-F | GGGAAAGTCGACCTTCCAGTGGTGCATGAACGC | SalI |
| hisG2-R | GGGAAACTGCAGGCTGTTCCAGTCAATCAGGGT | PstI |
| Ts-Ar-F | GGGAAACTGCAGAAGCCACTGAAGGTGGTCCA | PstI |
| Ts-Ar-R | GGGAAAGAGCTCAGCGTGCAAATCAGTGTGAC | SacI |
| Ts-Amr-F | GGGAAACTGCAGGATTTAGCTAATGTTGCTCC | PstI |
| Ts-Amr-R | GGGAAAGAGCTCGGAAAGAACCATCGTGGGT | SacI |
| ADH1SacI-F | GGGAAAGAGCTCTATGCATGCACAATTCTCAAAAT | SacI |
| ADH1KpnI-R | GGGAAAGGTACCTTATTTAGCAGTGTCCAAAACG | KpnI |
| ADH1Sal1-F | GGGAAAGTCGACATGCATGCACAATTCTCAAAAT | SalI |
| ADH1Xmal-R | GGGAAACCCGGGTTATTTAGCAGTGTCCAAAACG | SmaI |
| qPCR-ADH1-F | GTGCTGGTGGTGGTTTAGGT |  |
| qPCR-ADH1-R | CAACGTAAACTTCGGCACCC |  |
| qPCR-ACTIN-F | TGGTGATGGTGTTACCCACG |  |
| qPCR-ACTIN-R | GCGGTGGTGGAGAAAGTGTA |  |


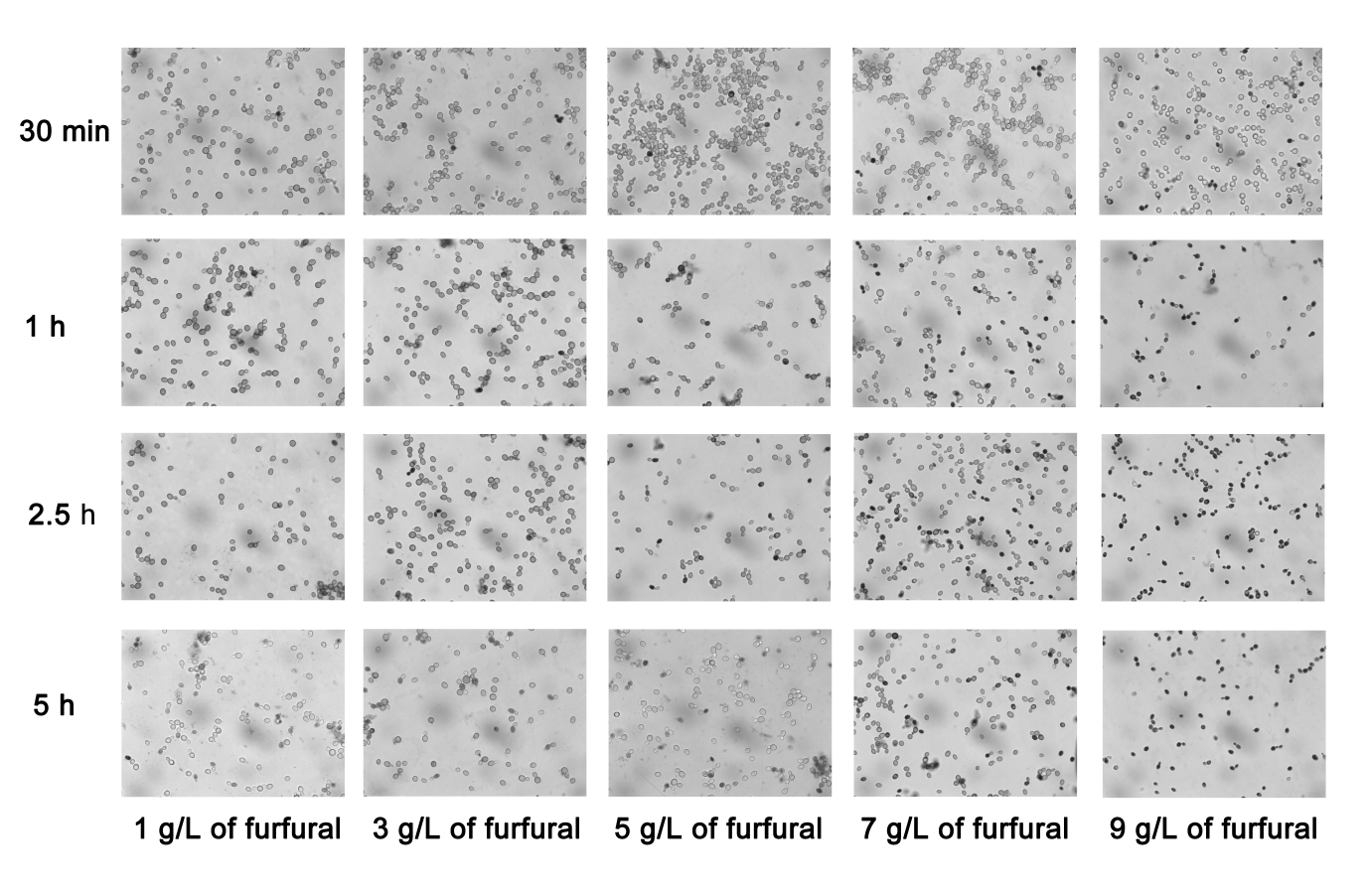


**Figure S1** **Furfural tolerance test evaluated by methylene blue staining.** Furfural of 1 g/L, 3 g/L, 5 g/L, 7 g/L and 9 g/L was added into the culture at middle exponential phase. After having been stained by Methylene blue solvent 30 min, cells were observed and photoed by optical microscope.


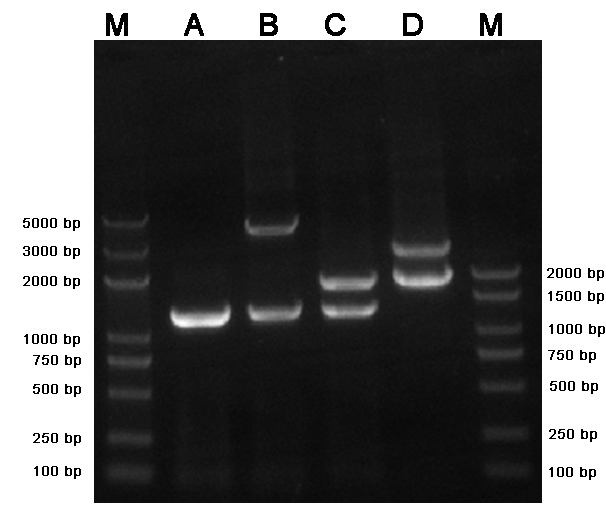


**Figure S2 PCR confirmation of the specific integration in sequential *ctADH1* disruption.** Lane M, DNA makers; Lane A, PCR from *C. tropicalis* YE genome with primers ADH1-F and ADH1-R resulting in the band of 1.2 kb (ADH1); Lane B, PCR from *C. tropicalis* Y1 genome with primers ADH1-F and ADH1-R resulting in the band of 1.2 kb and 4.3 kb (ADH1 and ADH1a-HisG-URA3-HisG-ADH1b); Lane C, PCR from *C. tropicalis* Y2 genome with primers ADH1-F and ADH1-R resulting in the band of 1.2 kb and 1.7 kb (ADH1 and ADH1a-HisG-ADH1b); Lane D, PCR from *C. tropicalis* Y4 genome with primers ADH1-F and ADH1-R resulting in the band of 1.7 kb and 2.9 kb (ADH1a-HisG-ADH1b and ADH1-URA3).

**
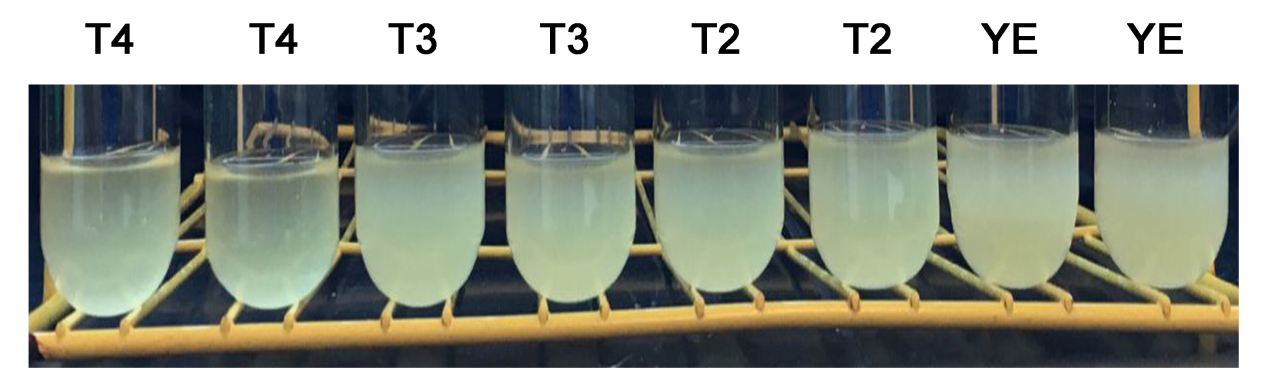
**

**Figure S3 Sensitivity experiment of *C. tropicalis* T4, T3, T2 and YE (parent strain).** Cells of *C. tropicalis* T4, T3, T2 and YE were inoculated into 5 ml YPD medium containing 3 g/L furfural. The plates were incubated for 10 h at 30 ^o^C.

**
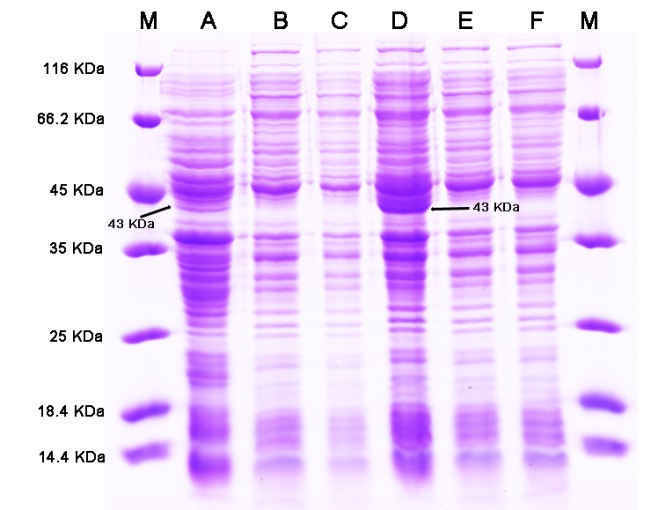
**

**Figure S4 SDS-PAGE of** **alcohol dehydrogenase 1 expressed in *E. coli*.** Lane M, protein molecular weight markers (Thermo Scientific, #26610, USA); Lane A, *E. coli* PCA cells after IPTG induction; Lane B, *E. coli* PCA cells before IPTG induction; Lane C, *E. coli* PC cells without IPTG induction; Lane D, *E. coli* PEA cells after IPTG induction; Lane E, *E. coli* PEA cells before IPTG induction; Lane F, *E. coli* PE cells without IPTG induction. Molecular weight of alcohol dehydrogenase 1 is around 43kDa.


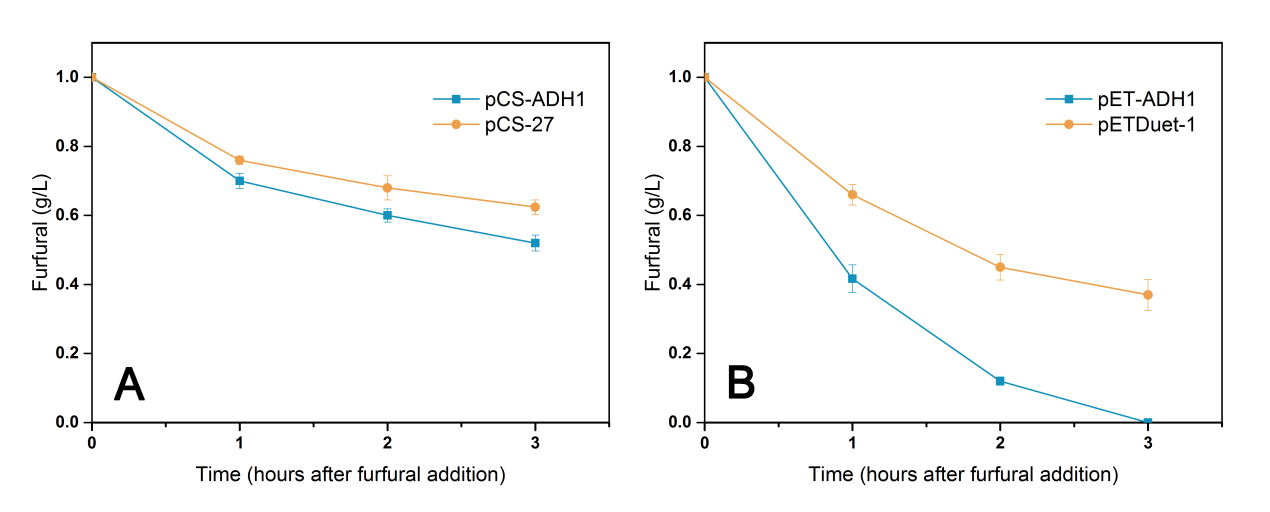


**Figure S5 In vivo furfural degradation in** **recombined** ***E. coli*.** (A) Furfural degradation of *E. coli* PCA (with pCS-ADH1) and PC (with pCS-27) in M9 medium; (B) Furfural degradation of *E. coli* PEA (with pET-ADH1) and PE (with pETDuet-1) in M9 medium.

1. Lin Y, Shen X, Yuan Q, Yan Y. Microbial biosynthesis of the anticoagulant precursor 4-hydroxycoumarin. Nature communications. 2013; 4.
